# A comparative study on chitosan nanoparticle synthesis methodologies for application in aquaculture through toxicity studies

**DOI:** 10.1049/nbt2.12047

**Published:** 2021-03-18

**Authors:** Subashni Bhoopathy, Dhinakaraswamy Inbakandan, Rajendran Thirugnanasambandam, Chandrasekaran Kumar, Pavithra Sampath, Ramalingam Bethunaickan, Vasantharaja Raguraman, Ganesh Kumar Vijayakumar

**Affiliations:** ^1^ Centre for Ocean Research (DST‐FIST Sponsored Centre) MoES—Earth Science & Technology Cell (Marine Biotechnological Studies) Col. Dr. Jeppiaar Research Park Sathyabama Institute of Science and Technology Chennai India; ^2^ Department of Immunology National Institute for Research in Tuberculosis Chennai India

## Abstract

Chitosan nanoparticles (CSNPs) have been recently used for various applications in aquaculture, especially as drug carriers. The aim of this study was to synthesise and investigate a superlative method of CSNP synthesis for application in aquaculture through aquaculture‐based toxicology screening methods. Two different methods were analysed: the first a direct ionic gelation method (A) and the other involving a low‐molecular‐weight chitosan microparticle intermediate method (B). Dynamic light scattering characterisation revealed that the CSNP particle sizes were 192.7 ± 11.8 and 22.9 nm from methods A and B, respectively. The LC_50_ values for brine shrimp toxicity were found to be 1.51 and 0.02 ppt in 24 h for methods A and B, respectively. Acute toxicity studies in *Litopenaeus vannamei* rendered LC_50_ values of 3235.94 and 2884.03 ppt in 24 h for methods A and B, respectively. Zebrafish toxicity studies revealed mortality rates of 21.67% and 55% at 20 mg/L concentration for methods A and B, respectively, with an increased expression of intracellular reactive oxygen species in method B. From these findings, it can be concluded that a comparatively reduced toxicity of CSNPs derived from ionic gelation method makes it more appropriate for application in aquaculture.

## 1 INTRODUCTION

Chitosan (CS) is a biodegradable compound, obtained from the deacetylation of N‐acetylglucosamine subunits of the polysaccharide chitin; CS is widely used as a drug‐delivery agent [1], and is applied in various fields such as the cosmetics, biomedical, and food industries, including aquaculture [2]. Its biocompatibility renders an enhanced membrane permeability property, which therefore makes it a propitious drug‐delivery agent, principally in the form of nanoparticles (NPs) [3].

Chitosan nanoparticles (CSNPs) are favourable drug carriers, especially for DNA vaccines and supplements, such as RNA nucleotides, vitamin C, hormones, etc. Oral administration of CSNPs has been shown to possess immune‐inducing activities in the field of aquaculture [4]. CSNPs have also been efficiently used in the form of films for preserving the fillets of sardine, croaker, salmon and trout [5, 6, 7, 8]. In aquaculture, CSNPs has also been used to deliver biological molecules and vaccines, orally or parenterally, in several organisms, specifically in *Clarias magur*, *Litopenaeus vannamei*, *Labeo rohita*, *Solea senegalensis*, and *Lates calcarifer* [3, 9, 10, 11, 12].

CSNPs are prepared by several methods, the most widely used being ionotropic gelation, polyelectrolyte complex, emulsification solvent diffusion, reverse micellar method, and microemulsion [13]. Among these methods, ionic or ionotropic gelation is most commonly used due to its low‐energy requirement and the non‐toxic nature of the raw materials utilised in the synthesis process [14]. The synthesis process involves cross‐linking of CS to poly‐anionic sodium tripolyphosphate (NaTPP) to form CS‐TPP NPs. Ionic gelation synthesis yields CSNPs within a size range of 100–300 nm, dependent on several factors like temperature, pH, concentration of CS and NaTPP, degree of deacetylation, and molecular weight of the CS [15, 16]. Some literature prefers a pre‐synthesis process prior to ionic gelation, involving a digestion process using chemicals such as sodium nitrite, in order to obtain more uniform smaller sized NPs by using a low‐molecular‐weight chitosan (LMWCS) microparticle intermediate [16, 17]. Although the application of CSNPs in aquaculture is extremely widespread, very limited literature on the toxicity of NPs in aquatic organisms is available.

The aim herein is to synthesise and investigate a superlative method for the synthesis of CSNPs: ionic gelation versus synthesis using a LMWCS microparticle intermediate through aquatic models and oxidative stress analysis. An extensive study of aquatic animals will provide an explicit appreciation of the toxic effects of CSNPs on aquatic organisms, which should be contemplated while applying these potential drug carriers in aquaculture.

## 2 MATERIALS AND METHODS

### 2.1 Materials

Glacial acetic acid (GAA) and sodium nitrite were acquired from Merck. CS (degree of deacetylation 85%, and viscosity 20–300 cP) was purchased from Sigma Aldrich. The dialysis membrane (MWCO: 3500) was obtained from Sigma. Ethanol and Luria–Bertani (LB) broth were procured from Himedia Laboratories Pvt. Ltd. India. Brine shrimp cysts were purchased from INVE Aquaculture Inc.,.

### 2.2 Synthesis of CS/TPP nanoparticles

The synthesis of NPs was carried out using two methods: (i) ionic gelation method (method A) and (ii) LMWCS intermediate method involving two steps, firstly high‐molecular‐weight CS was degraded to LMWCS and this was followed by an ionic gelation process to obtain CSNPs (method B).

#### 2.2.1 Ionic gelation method (method A)

CSNPs were prepared by ionic gelation process using the method of Duse et al. [18] with slight modifications. CS solution was prepared by dissolving CS at a concentration of 3 mg/ml in 0.5% acetic acid solution. NaTPP was dissolved in deionised water at a concentration of 1 mg/ml. NaTPP was added to chitosan solution with steady stirring, until an opalescent white solution was obtained, which was centrifuged at 16,000 rpm for 15 min. The supernatant was aspirated and the pellet containing the NPs was collected. The pellet was consequently washed with 20%, 75%, and 100% ethanol, respectively, and freeze‐dried for further use.

#### 2.2.2 Low‐molecular‐weight chitosan intermediate method (method B)

In this method, CSNPs were synthesised using two steps. Firstly, LMWCS were synthesised by unspecific digestion of CS polymers. Secondly, the digested LMWCS oligomers were segregated and subjected to ionic gelation to form CSNPs. This synthesis of LMWCS was adapted from Kunjachan et al. [16]. CS solution (3 mg/ml) was prepared using 0.5% GAA. Sodium nitrite (1% w/v) was added to CS solution and incubated in 150 rpm orbital shaker at 37°C for 1 week. This solution was subjected to dialysis (MWCO: 3500) overnight against deionised water. The dialysis membrane retained uniformly sized oligomers and allowed water‐soluble monomers to drift through. The obtained LMWCS was subjected to ionic gelation. LMWCS solution was prepared by dissolving 1% (v/v) LMWCS in 1% GAA solution. NaTPP (10% w/v) was dripped into 5 ml LMWCS until an opalescent white solution was obtained. On the addition of NaTPP, the pH shifted towards alkaline (≥7.5), and it was adjusted to acidic pH (4.5–5.0) using 1 M HCl. The solution was centrifuged at 7500 rpm, for 15 min at 14°C. The pellet was subsequently washed with 20%, 75%, and 100% ethanol, respectively, to remove excess NaTPP, and was further lyophilised for analysis.

### 2.3 Characterisation of chitosan nanoparticles

#### 2.3.1 Atomic force microscopy (AFM)

The synthesised CSNPs were scanned using AFM to evaluate their morphology. CSNPs were dissolved in 0.5% GAA and sonicated at 30 KHz for 15 min using Rivotek Ultrasonic Cleaner. The sonicated suspensions were spread as a thin layer evenly on a glass slide, air dried, and analysed using an AFM (NTEGRA Prima), having silicon nitride cantilevers with 3.4 × 1.6 × 0.3 mm dimensions, 6 nm tip curvature radius, and a force constant of 25 N/m. The images were analysed using Nova^TM^ software (NT‐MDT, Spectrum instruments).

#### 2.3.2 Measurement of zeta potential and particle size

The zeta potential and mean particle diameter of the CSNPs were discerned using an electrophoretic light scattering and dynamic light scattering (DLS) device (Horiba SZ‐100—NanoparticaSeriesInstruments, Kyoto, Japan).

### 2.4 Toxicity studies

#### 2.4.1 Cytotoxicity

Thiazolyl blue tetrazolium bromide (MTT) assay was performed to evaluate the relative cytotoxicity of the CSNPs on chick embryo fibroblast (CEF) cells. The cells were seeded in a 96‐well plate at the rate of 15,000 cells/well using 100 μL of culture medium. After 48 h, the spent medium was removed and replaced with 100 μL of media containing NPs between the range of 0.001% to 1% for 4 and 24 h. Following this, the medium was removed and washed with 100 μl of 0.1 M phosphate‐buffered saline (PBS), before adding 100 μl of 1 mg/ml MTT. After 30 min of incubation, the MTT solution was removed, washed with 0.1 M PBS, and 100 μl DMSO was added. The absorbance was measured at 570 nm following 30 min incubation.

#### 2.4.2 Brine shrimp lethality assay

The protocal of Ashtari et al. [19] was followed with slight modifications for carrying out the brine shrimp lethality assay. Cysts of *Artemia salina* were added to well‐aerated tanks containing sterile seawater (35 ppt) and kept under continuous illumination for about 28–30 h at room temperature. The hatched nauplii were collected and used for the experiments. The synthesised CSNPs were experimented in various concentrations ranging between 0.001% and 1% on brine shrimps. The experiment was performed in triplicate with 10 larvae in each beaker. To facilitate aeration, the beakers were placed in an orbital shaker (Lark refrigerated shaker) at 8 rpm at room temperature. The mortality rate was recorded after 24 h. Gallic acid at 20 µg/ml was used as a positive control. The LC_50_ value was calculated by probit analysis with 95% confidence limits.

#### 2.4.3 Acute toxicity in shrimps

Shrimps (*L. vannamei*) were purchased from a commercial hatchery in Kalpakkam, Chennai, at postlarval stage and acclimatised in the laboratory for 7 days. The shrimps were fed *ad libitum* and maintained under a 12:12‐h light–dark cycle with constant aeration in UV‐treated seawater. The acute toxicity experiments were performed based on the Standard Guide for Conducting Acute Tests with Fishes (EPA/ROC, 1998). The shrimps ,with an average size of 7.36 ± 1.82 cm, were selected and divided into nine groups (in triplicate) containing 10 shrimps each: a control group and eight groups for different concentrations (25, 50, 75, 100, 250, 500, 750, and 1000) for each type of CSNP. The mortality rate was recorded after 24, 48, 72, and 96 h, respectively. The dead shrimps were removed along with the excreta and a complete water exchange was done to ensure the absence of ammonium accumulations. The LC_50_ value was calculated by probit analysis with 95% confidence limits.

#### 2.4.4 Evaluation of toxicity in zebrafish

##### Exposure of zebrafish embryos to chitosan nanoparticles

Zebrafish (ZF) toxicity evaluation was carried out based on Hu et al. [20] with modifications. The male and female ZF (*Danio rerio*) were bred and the eggs collected, washed, and maintained in E3 medium. To assess the effect of the two different CSNPs, 20 embryos that had reached the blastula stage were exposed to different concentrations (5, 10, 20 mg/L) of CSNPs and incubated for 72 h at 28.5°C in triplicate. 1X E3 medium was used as a negative control and ZnO NPs (10 mg/L) was used as a positive control. After 96 h post‐fertilisation, the mortality and hatching rates were evaluated and the developmental malformations were observed and documented under a stereo microscope (Leica M205 A, Leica Microsystems). The mortality and hatching rates were expressed as the number of dead and hatched embryos, respectively, obtained 96 h post‐fertilisation, as compared to the control group.

##### Evaluation of intracellular reactive oxygen species in zebrafish embryos

NPs are known to cause reactive oxygen species‐mediated oxidative stress. In order to evaluate this, intracellular ROS was determined using flow cytometry by using a fluorescent probe 2′,7′‐dichlorodihydrofluorescein diacetate (H_2_DCFDA). The ROS reacts with the acetate group, leading to the conversion of non‐fluorescent H_2_DCFDA to a highly fluorescent 2′,7′‐dichlorofluorescein (DCF). About 30 hatched larvae were collected and exposed to the NPs. Hydrogen peroxide (10 nmol/L) was used as a positive control. A maximum volume of E3 medium was removed 96 h post‐fertilisation and replaced with sterile ice‐cold 1X PBS. The larvae were then homogenised after removing the PBS. The tubes were centrifuged at 5000 rpm for 5 min. The cells were washed once with 1X PBS, before adding 100 µl of FACS buffer (3% foetal bovine serum in PBS). The single‐cell suspension thus obtained was treated with the respective NPs along with 1.25 mg/L H_2_DCF‐DA and incubated at 28.5°C for 20 min in the absence of light. The tubes were then centrifuged and the pellet was washed with 1X PBS and resuspended in FACS buffer. The cells (10,000 events) were acquired with 480 nm blue laser excitation and analysed for signal detection at an excitation wavelength of ∼492–495 nm and emission wavelength of ∼517–527 nm under 510/20 nm bandpass filter using a flow cytometer (Becton Dickinson FACS Aria II, BD Biosciences). The analysis was performed using FlowJo software 10.0.1 (FlowJo LLC).

### 2.5 Statistical analysis

Statistical evaluation was performed using probit analysis and *t*‐test assuming equal variances in Microsoft® Office Excel® (version: 2007) and the results depicted as mean ± standard deviation, which were obtained from three independent experiments. A *p* value ≤ 0.05 was considered significant and *p* value ≤ 0.01 was considered statistically very significant, unless otherwise stated.

## 3 RESULTS AND DISCUSSION

### 3.1 Characterisation of chitosan nanoparticles

**FIGURE 1:**
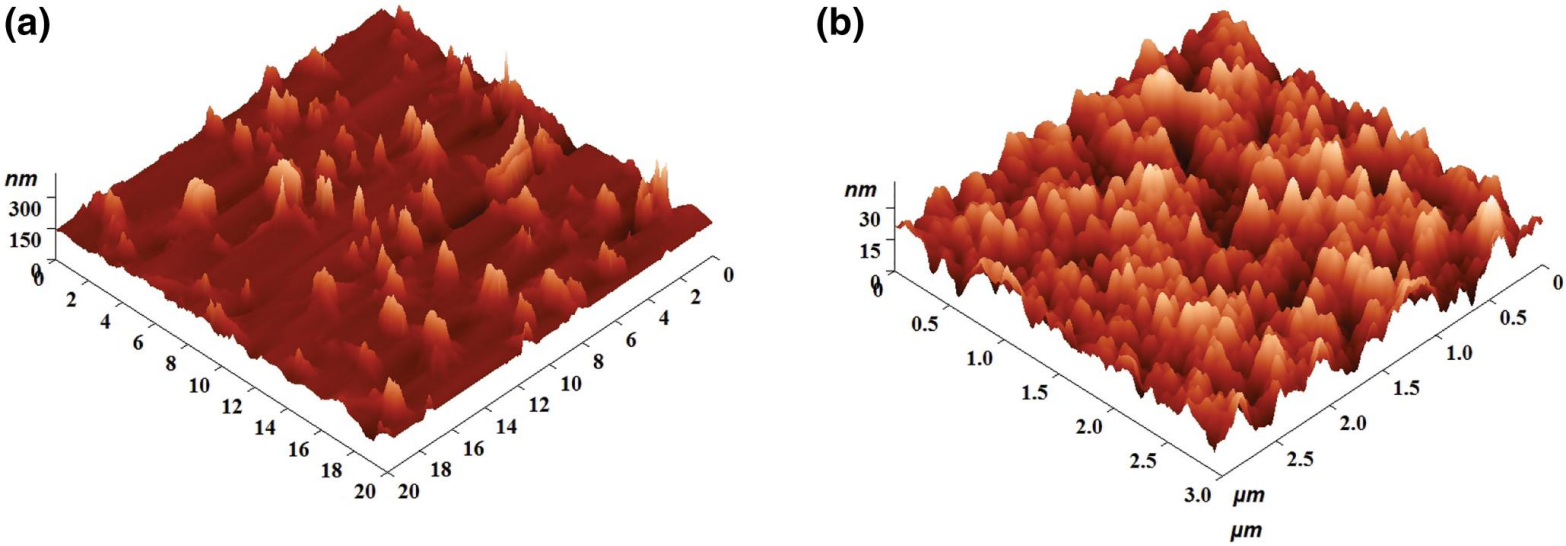
Atomic force microscopy images of (a) method A CSNPs showing asymmetrical NPs of 161.09 nm size, and (b) method B CSNPs showing symmetrical NPs of 20.05 nm size. CSNPs; chitosan nanoparticles; NPs, nanoparticles

**FIGURE 2:**
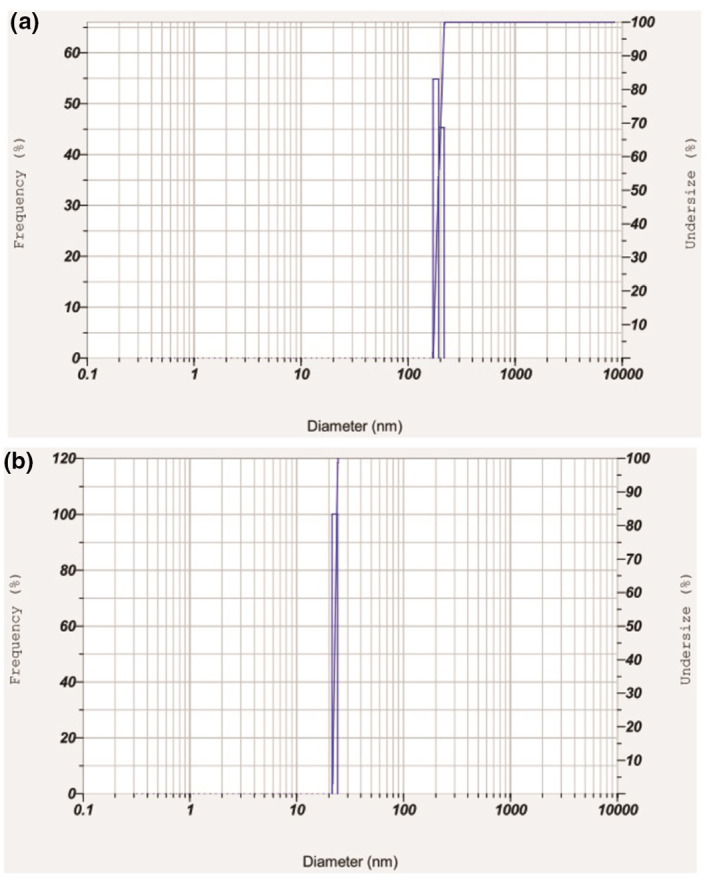
Dynamic light scattering analysis graph showing (a) method A CSNPs of 192.7 ± 11.8 nm size and (b) method B CSNPs of 22.9 nm size. CSNPs; chitosan nanoparticles

AFM analysis revealed the synthesis of asymmetrical NPs with a size of 161.09 nm (Figure 1a) from method A and symmetrical NPs of 20.05 nm (Figure 1b) diameter from method B. DLS analysis, similarly, manifested the synthesis of 192.7 ± 11.8 (Figure 2a) and 22.9 nm (Figure 2b) sized NPs from methods A and B, respectively. The molecular weight of CS is a major determining factor in regulating the particle size [16]. The degradation of CS to LMWCS was facilitated by sodium nitrite followed by dialysis, which triggered the synthesis of a more symmetrically shaped and smaller sized nanomaterial in method B as compared to method A.

Yousefpur et al. performed a similar linear depolymerisation reaction of CS using various concentrations of sodium nitrite solution and concluded that the depolymerisation of CS to LMWCS is highly dependent on the molar ratio of glucosamine subunits to sodium nitrite [17]. The utilisation of CS, without depolymerisation, yields larger sized NPs of 250–400 nm in the ionic gelation process of CSNP synthesis [21, 22]. Hydrogen peroxide‐mediated degradation of CS is also not an uncommonly used temperature‐dependent digestion technique. Zhao et al. showed that hydrogen peroxide‐mediated degradation of CS engendered depolymerised CS with molecular weight over 1.0 × 10^6^ and 5.0 × 10^5^, which in turn yielded NPs larger than 100 nm and smaller than 20 nm, respectively, during ionic gelation using an aqueous TPP solution [23]. However, it should be discerned that sundry factors of CS such as degree of deacetylation, concentration, and degree of cross‐linking with TPP during ionic gelation also impact the average hydrodynamic diameter of the resultant NPs [14].

### 3.2 Toxicity studies

#### 3.2.1 Cytotoxicity

The cytotoxicity of CEF cells was found to be significantly higher in method B in comparison with method A during a 24 h exposure period. A significant difference in the viability of cells, namely 81.98% and 48%, was observed in methods A and B, respectively, at 0.05% concentration.

**FIGURE 3:**
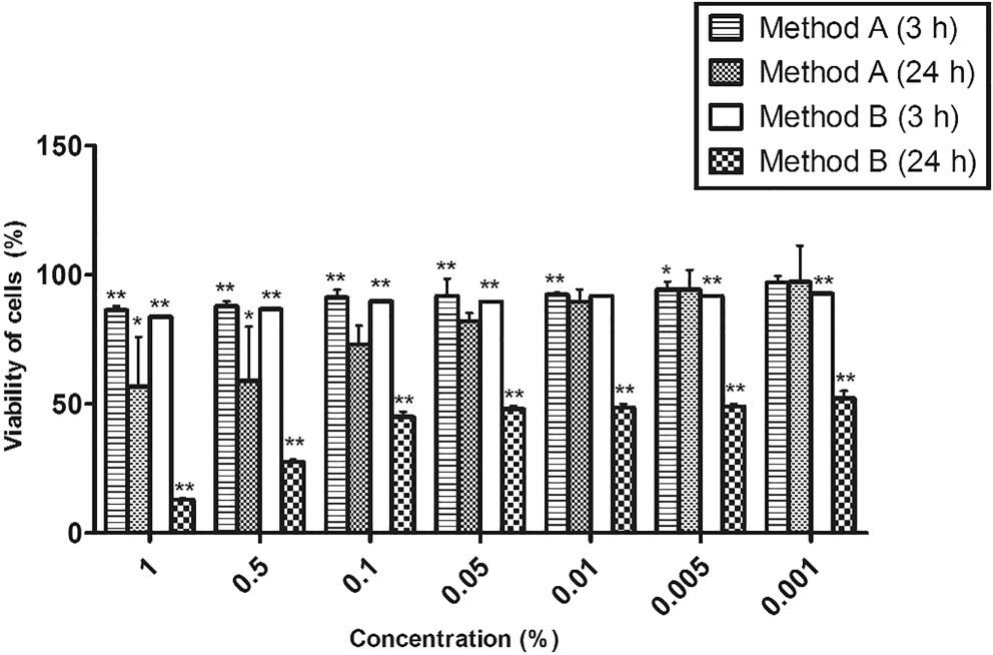
Cytotoxicity of chick embryo fibroblast cells exposed to methods A and B CSNPs for 3 and 24 h. Significant differences in the % viability of cells was observed in the cells exposed to both methods A and B CSNPs for 24 h. Among these, method A CSNPs had higher % viability compared to method B CSNPs. *p value ≤ 0.05; **p value ≤ 0.01. CSNPs; chitosan nanoparticles

However, in 3 h exposure the viability was higher than 80% in both types of NPs (Figure 3). It can be implied that a decrease in the size of the CSNPs causes an increase in the cytotoxicity; therefore suggesting that method B CSNPs are more toxic than method A CSNPs. The viability rate of SISK cells was found to be above 90% for a concentration range between 1 and 5 mg/ml of the CSNPs (30–60 nm in size), that were synthesised by the ionic gelation method [3]. The % viability of the BEL7402 cell line decreased with a decrease in the particle size (100 > 70 > 40 nm). Also, the IC_50_ values of CSNPs with 40, 70, and 100 nm hydrodynamic diameters were evaluated to be 14.98, 16.54, and 23.84 µg/ml respectively, stipulating that a decrease in size leads to an increase in cytotoxicity, due to an increased accumulation of NPs in cells [24].

CSNPs exhibit cytotoxicity due to increased Reactive oxygen species (ROS) generation, which produces an oxidative stress state in the target organism. A disturbance in the physiological redox state results in the production of free radicals that rapidly reacts with DNA, proteins, and lipids, causing increased cellular damage. According to the hierarchical model of oxidative stress, upon exposure of an organism to NP‐mediated oxidative stress, it combats this using an antioxidant enzyme system. However, cell death occurs when an intolerable amount of oxidative stress is caused due to an increased amount of ROS, which in turn causes mitochondrial membrane damage and other oxidative scarring including DNA damage and activation of signalling pathways that are associated with a loss of cell growth [25].

#### 3.2.2 Brine shrimp lethality assay

**FIGURE 4:**
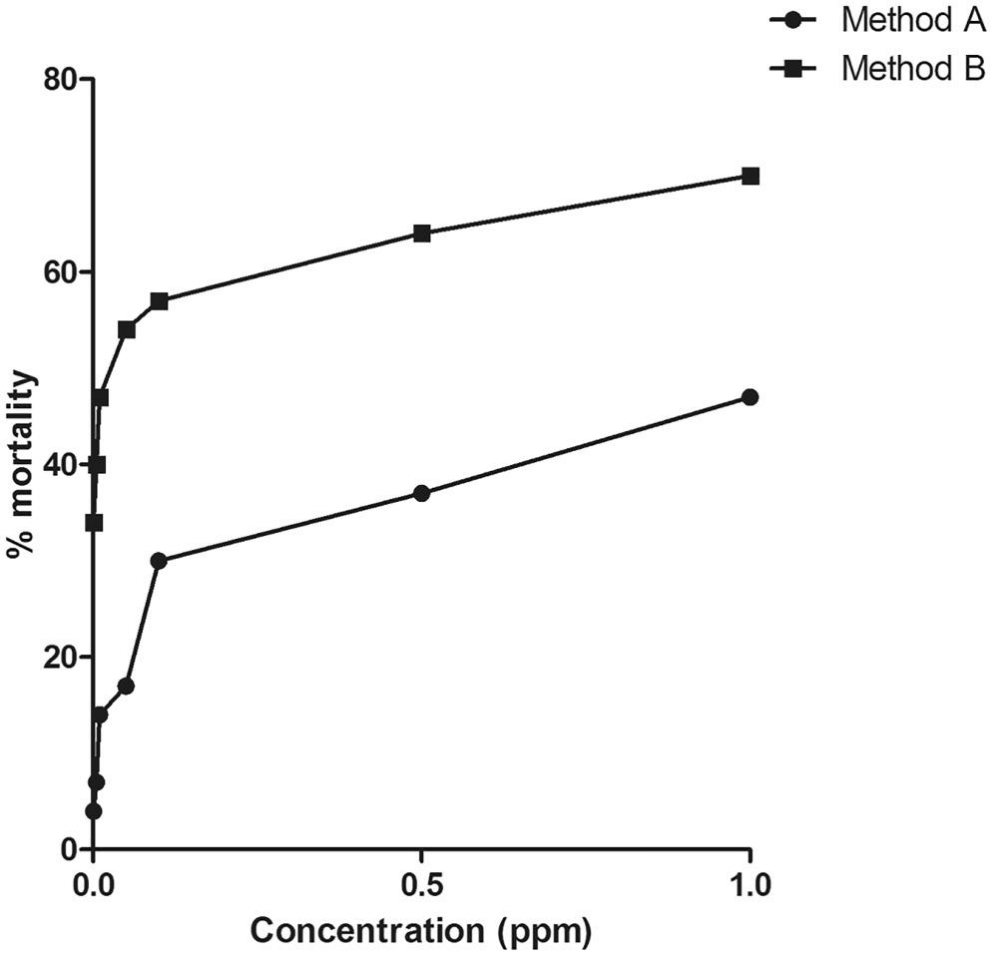
Graph depicting the differences in the mortality rate of the *Artemia salina* exposed to methods A and B CSNPs. The LC_50_ values of method A and B CSNPs on the brine shrimps were found to be 1.51 and 0.02 ppm, respectively. CSNPs; chitosan nanoparticles

The exposure to brine shrimp nauplii larvae showed significant differences in the LC_50_ values between both types of CSNPs. The LC_50_ values were found to be 1.51 and 0.02 ppm for the CSNPs synthesised by methods A and B, respectively, at 24 h exposure. As evident from Figure 4, the % mortality was found to be appreciably higher in method B CSNPs than method A CSNPs. However, the cytotoxicity of CS was found to vary between fresh and salt water media, a decrease in the salinity caused an increase in the cytotoxicity, which can be attributed to the inflation of membrane penetration, due to the breach in ionic homoeostasis [26].

The toxicity of the two types of CSNPs will therefore differ at a larger range in fresh and marine aquatic applications. Nevertheless, it should be discerned that the gross toxicity of the CSNPs may vary depending on the conjugate/drug employed in target/drug delivery. The effect of CS on the lethality and cell growth of *Artemia* spp. is highly influenced by the molar mass of CS [26].

#### 3.2.3 Acute toxicity in shrimps

**FIGURE 5:**
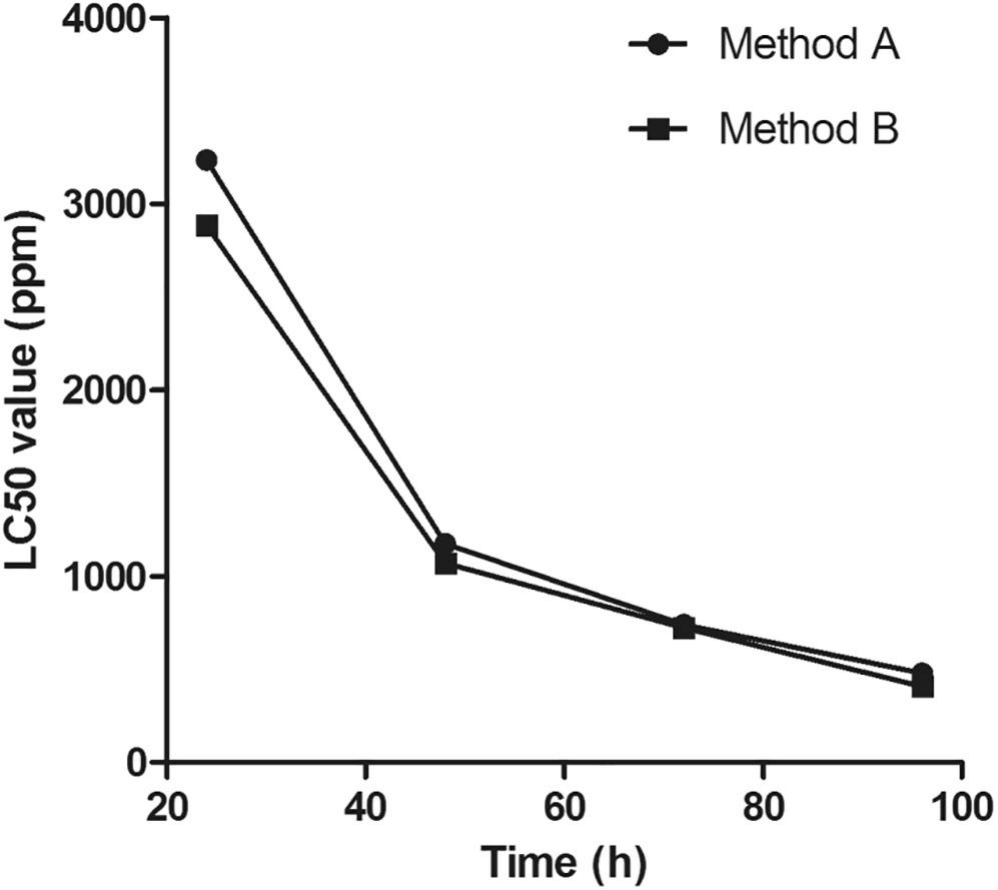
Graph depicting the differences in the LC_50_ of the *Litopenaeus vannamei* exposed to methods A and B chitosan nanoparticles

The acute toxicity analysis of CSNPs on post‐larval *L. vannamei* shows a prominent difference between the LC_50_ values for the first 24 h. As can be seen from Figure 5, post‐48 h, the difference between the LC_50_ values of the CSNPs synthesised from methods A and B was found to abate. The acquired LC_50_ values were as follows: 3235.94 and 2884.03 (24 h); 1174.9 and 1071.52 (48 h); 741.31 and 724.44 (72 h); 478.63 and 407.38 (96 h) for methods A and B, respectively. The abatement in the differences of the LC_50_ values could be explained by the increased toxicity caused by the method A CSNPs due to a prolonged exposure period. Hence a time‐dependent and dose‐dependent increase in the toxicity of CSNPs can be conclusive from the findings of this experiment. Furthermore, the CSNPs were not orally administered via feed to the shrimps; rather, they were dispersed in the water containing the shrimps. These nanoaggregates could possibly amass in the gills of postlarval shrimps. For the aforementioned rationale, these LC_50_ values may be slightly ambiguous, while considering the application of CSNPs for oral drug delivery. Wu and Chen demonstrated that the acute toxicity of Cd and Zn on *L. vannamei* affects the oxygen consumption, ammonium excretion, and metal accumulation within the gills [27].

#### 3.2.4 Evaluation of toxicity in zebrafish

##### Exposure of zebrafish embryos to chitosan nanoparticles

**FIGURE 6:**
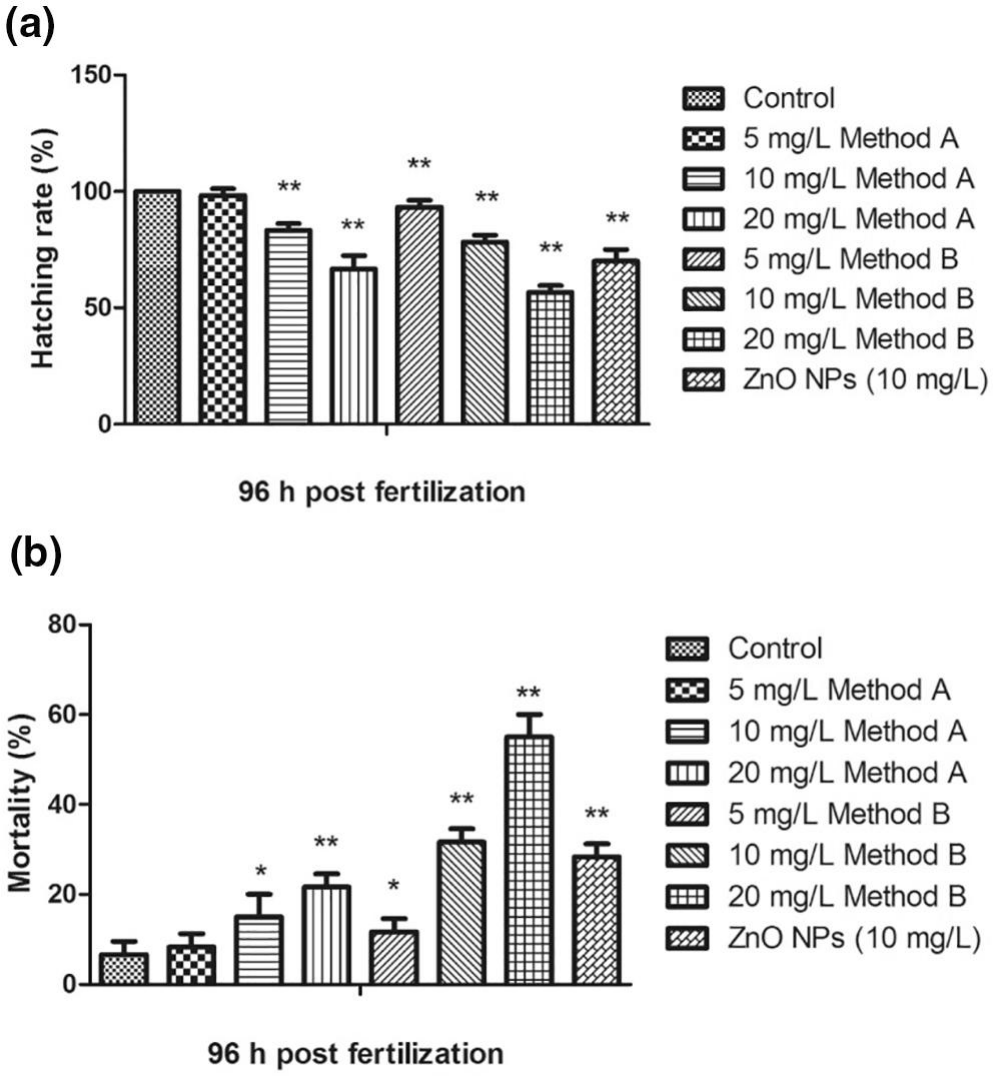
Zebrafish studies representing differences in the (a) hatching and (b) mortality rates were observed for the cells exposed to methods A and B chitosan nanoparticles. *p value ≤ 0.05; **p value ≤ 0.01

Differences in the hatching and mortality rates along with the developmental abnormalities were observed in the CSNPs synthesised using both methods. The differences in the hatching rate of the ZF embryos (Figure 6a )exposed to 10 and 20 mg/L concentrations of method A CSNPs and 5, 10, and 20 mg/L concentrations of method B CSNPs were found to be statistically very significant (*p* ≤ 0.01), compared with the control. However, a significant increase (*p* ≤ 0.05) in the mortality rate of the ZF embryos (Figure 6b), exposed to 5 mg/L concentration of method B CSNPs and 10 mg/L concentration of method A CSNPs was observed. Furthermore, a very significant (*p* ≤ 0.01) increase in the mortality rate of the ZF embryos exposed to 20 mg/L method A CSNPs and 10 and 20 mg/L method B CSNPs was observed.

**FIGURE 7:**
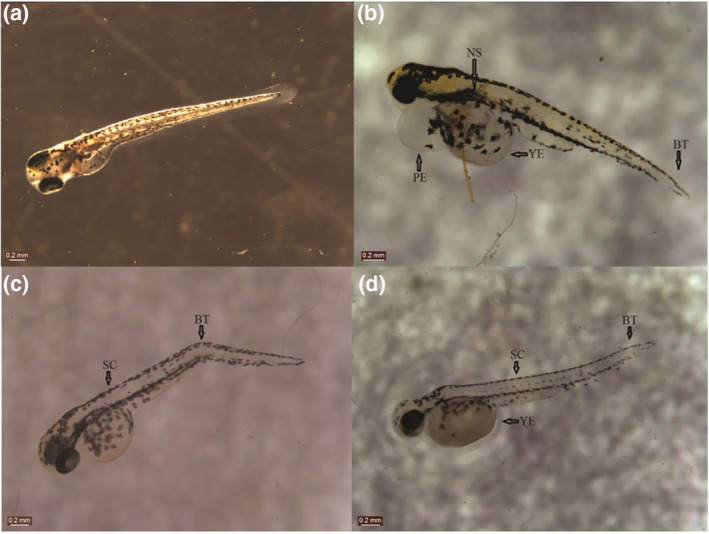
Zebrafish embryos showing malformations compared to those of (a) controls. ZF embryos depicting various malformations were documented: (b) NS, BT, PE, YE; (c) SC, BT; (d) SC, BT, YE. The rate of malformation was independent of dose and the ZF embryos exposed to both NPs exhibited some degree of malformation. BT, bent‐tail; NPs, nanoparticles; NS, non‐inflated swim bladder; PE, pericardial oedema; SC, spinal curvature; YE, yolk‐sac oedema; ZF, Zebrafish

The toxicity of the CSNPs was dose‐dependent and also the toxicity of method B CSNPs was higher than method A CSNPs, as evident from the hatching and mortality rates. Additionally, several (not numerically well‐defined) ZF embryos, that were experimented in 10 mg/L and 20 mg/L concentrations of both the CSNPs exhibited malformations (Figure 7), such as spinal curvature (SC), pericardial oedema, bent tail, non‐inflated swim‐bladder, and yolk sac oedema besides the decreased hatching rate. The occurrence in the number of embryos displaying these malformations was, however, independent of the concentration.

ZF embryos have been demonstrated to be excellent models for toxicity studies, especially in aquaculture. Hu et al. reported dose‐dependent and time‐dependent differences in the mortality rate and a dose‐dependent decrease in the hatching rate of the ZF embryos exposed to CSNPs [20]. The ZF embryos that were exposed to 200 nm sized CSNPs (starting from 5 mg/L concentration) possessed developmental abnormalities and the rate of malformation was concentration‐dependent; no significant malformations were observed in the ZF embryos exposed to 340 nm CSNPs [20]. Biodegradable CSNPs (size: 84.86 nm) and conventional CSNPs were shown to possess toxicity in ZF embryos with developmental abnormalities and a reduced hatching rate [28]. CSNPs, at lower concentration (5 µg/ml), were found to bolster the immune response of ZF embryos, against the pathogen *Aeromonas hydrophila*; however, the toxicity of CSNPs remains at higher doses [29]. The toxicities of various other nanomaterials such as ZnO NPs, copper NPs, and carbon nanotubes have been studied in ZF embryos and have significantly demonstrated increased mortality, decreased hatching rate, along with developmental malformations [30, 31, 32].

##### Evaluation of intracellular reactive oxygen species in zebrafish embryos

**FIGURE 8:**
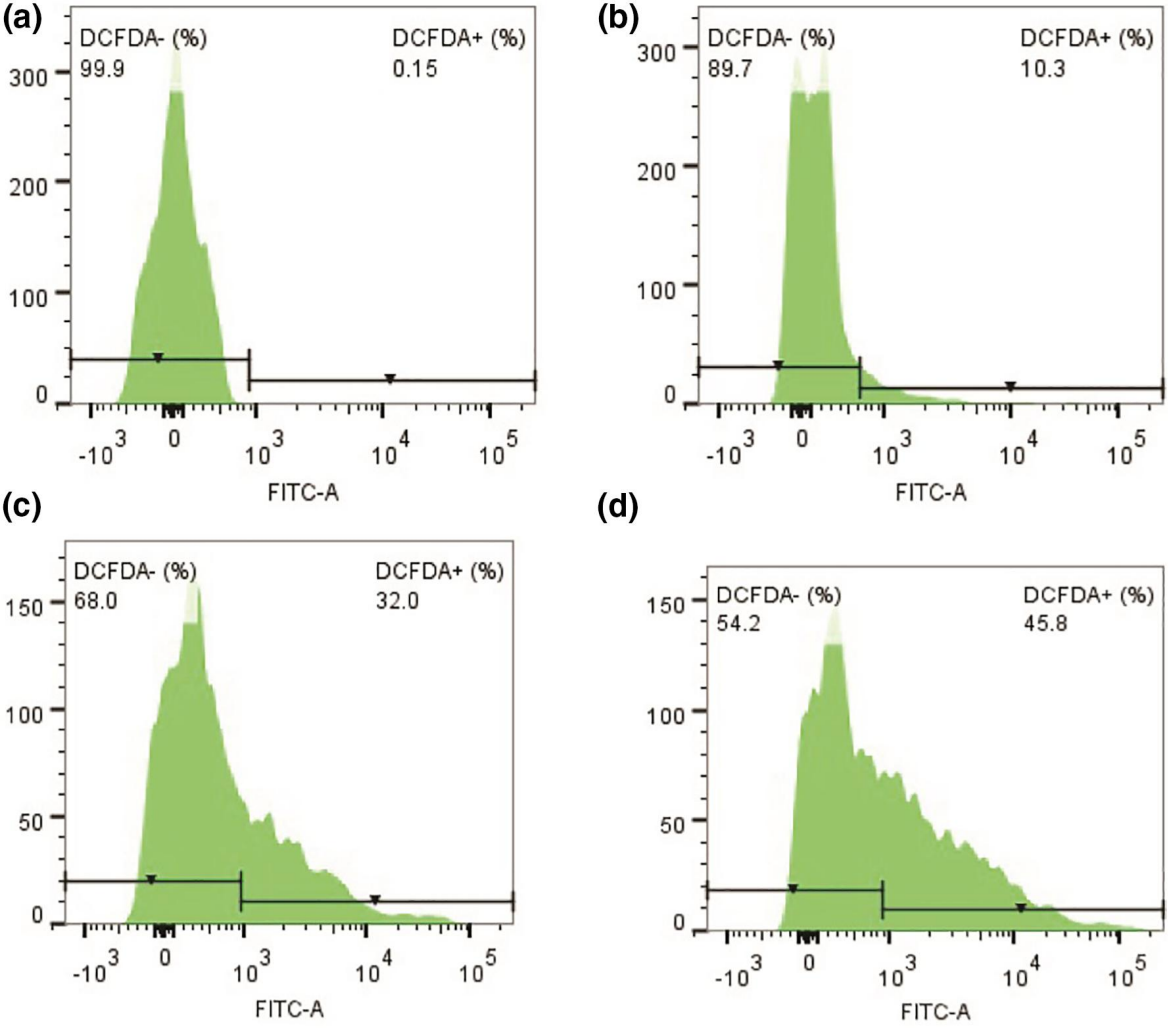
Flow cytometry histogram representation of the cell suspension from the zebra fish exposed to (a) control; (b) 10 mg method B CSNPs; (c) 20 mg method B CSNPs; and (d) H_2_O_2_ (10 nmol/L). DCFDA, dichlorodihydrofluorescein diacetate; CSNFs, chitosan nanoparticles

**FIGURE 9:**
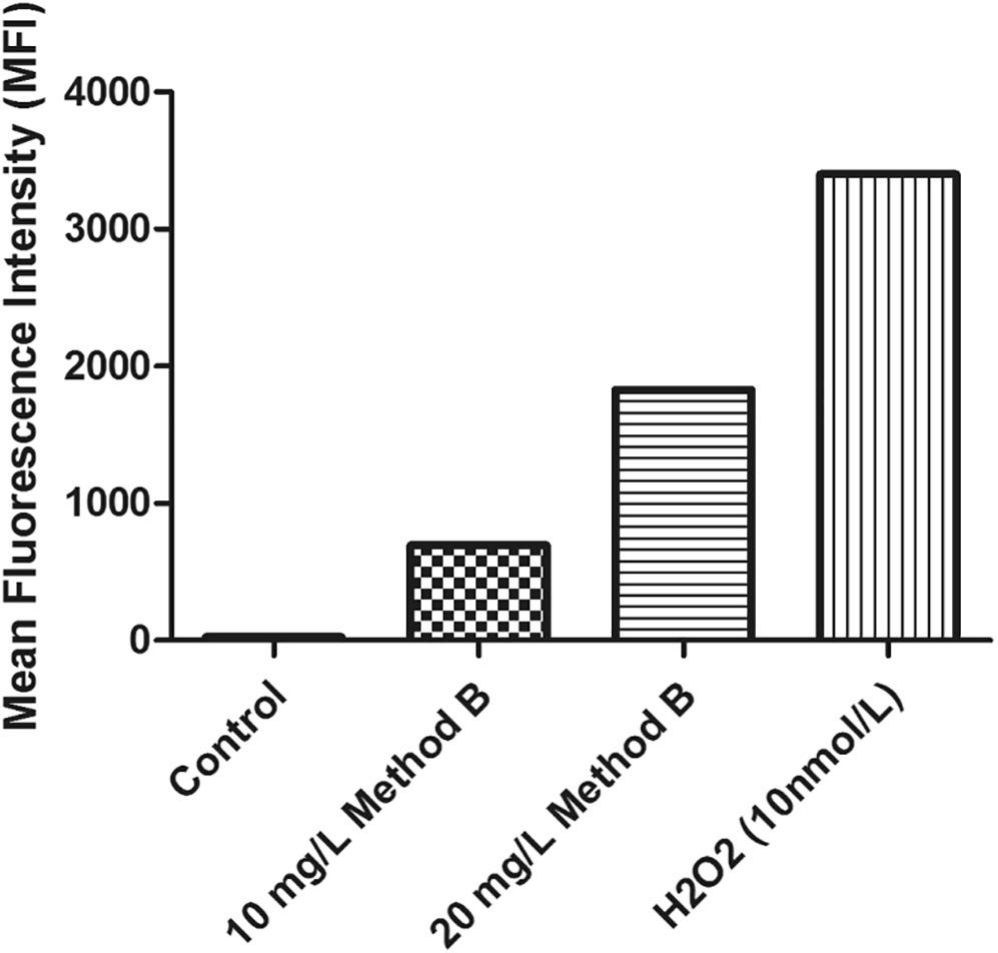
The mean fluorescent intensity of the corresponding cells positive for dichlorodihydrofluorescein diacetate along with control and H_2_O_2_ (10 nmol/L) exposed ZF embryonic cells

The intracellular ROS analysis in the ZF embryos (Figure 8) indicated a significant increase in the percentage of positive cells for DCFDA expression in the cell suspension exposed to 10 mg/L (10.3%) and 20 mg/L (32%) concentrations of method B CSNPs, compared to that of the control cells (0.15%). The degree of DCFDA expression is also evident from the mean fluorescent intensity of the cells producing ROS (Figure 9). However, no significant difference in the DCFDA signal from the other cells was observed.

Nanomaterial, being a xenobiotic, acts as an exogenous inducer and produces immune reaction in the administered host organism [33]; the production of ROS is one such immune response. The most predominant form of ROS, O_2_^.‐^ is generated by nicotinamide adenine dinucleotide phosphate oxidase using molecular oxygen due to the interaction of NP with various biological targets. An increased amount of ROS production, coupled with the inability of the organisms to detoxify the resulting intermediate compounds, leads to oxidative stress [25]. An increase in the ROS was hypothesised to be associated with the incidence of developmental toxic effects and high mortality rate at higher concentrations. Moreover, a significant increase in the intracellular ROS of the ZF embryonic cells has been reported in 5 mg/L concentration of CSNPs [19]. In another study, TiO_2_ NPs caused ROS‐mediated toxicity in ZF larvae by absorbing photons [34].

## 4 CONCLUSION

To summarise, the toxicological studies including acute toxicity experiments on *A. salina* and *L. vannamei* and the toxicity studies on ZF embryos, all indicate significantly higher toxicity of method B CSNPs compared to that of method A CSNPs. The remnant of digestion chemical (sodium nitrite) and the smaller size (hypothetically, causing an increased penetration) of the CSNPs synthesised from method B may render these NPs with a relatively elevated toxicity. The findings suggest that an undemanding ionic gelation method A is more befitting for the CSNP synthesis to be applied in the field of aquaculture; it should also be noted that it is the most widely used method of CSNP synthesis. Although smaller sized CSNPs synthesised from LMWCS intermediates have increased toxicity, it should also be considered that the ZF embryos exposed to both types of CSNPs displayed some degree of malformation. Therefore, a meticulous examination on the effect of CSNPs, along with the respective conjugate/drug in the target aquatic organism, should be thoroughly studied, before introducing these indispensable drug‐delivery agents in aquaculture farms.

## Supporting information

Supplementary Material 1
