# Supplementary material for: A comparative study on chitosan nanoparticle synthesis methodologies for application in aquaculture through toxicity studies
Source: IET Nanobiotechnol. 2021 Mar 18;15(4):418–26. doi: 10.1049/nbt2.12047 (PMC8675861; doi:10.1049/nbt2.12047)
Supplement: Supplementary file 1 — Supplementary Material 1 [file NBT2-15-418-s001.docx]

**S1. Table illustrating the differences in the mortality rate of the *Artemia salina* exposed to method A and B CSNPs.**

| Concentration (ppm) | Mortality rate in Method A CSNPs (%) | Mortality rate in Method B CSNPs (%) |
| --- | --- | --- |
| 1 | 47 | 70 |
| 0.5 | 37 | 64 |
| 0.1 | 30 | 57 |
| 0.05 | 17 | 54 |
| 0.01 | 14 | 47 |
| 0.005 | 7 | 40 |
| 0.001 | 4 | 34 |

**S2. Table illustrating the differences in the LC_50_ of the *Litopennaeus vannamei* exposed to method A and B CSNPs.**

| Time of exposure (h) | LC_50_ value for Method A | LC_50_ value for Method B |
| --- | --- | --- |
| 24 | 3235.94 | 2884.03 |
| 48 | 1174.9 | 1071.52 |
| 72 | 741.31 | 724.44 |
| 96 | 478.63 | 407.38 |
